# Trends in novel antiandrogen receptor signal inhibitor use and medical costs in prostate cancer

**DOI:** 10.1002/cam4.70226

**Published:** 2024-12-15

**Authors:** Hikari Miura, Hayato Yamamoto, Yoshiharu Okuyama, Noritaka Ishi, Ryuma Tanaka, Takuya Oishi, Fumiya Yoneyama, Tomoko Hamaya, Kyo Togashi, Naoki Fujita, Teppei Okamoto, Chikara Ohyama, Shingo Hatakeyama

**Affiliations:** ^1^ Department of Urology Hirosaki University School of Medicine Hirosaki Japan; ^2^ Department of Advanced Transplant and Regenerative Medicine Hirosaki University School of Medicine Hirosaki Japan

**Keywords:** ARSI, cost, NDB, prostate cancer, use

## Abstract

**Objective:**

We aimed to examine trends in novel antiandrogen receptor signal inhibitor (ARSI) usage and medical costs by collecting real‐world big data included in The National Database of Health Insurance Claims and Specific Health Checkups of Japan (NDB) Open Data, covering most of the clinical practices throughout Japan.

**Methods:**

Usage data for outpatient prescriptions from 2016 to 2021 were extracted from the NDB Open Data. Among the 459,610 million tablets/capsules prescribed, prostate cancer‐specific agents (bicalutamide, estramustine phosphate, flutamide, abiraterone, enzalutamide, apalutamide, and darolutamide) were selected to investigate the trends of usage and medical costs.

**Results:**

In total, 764.8 billion medications were recorded. Among these, standard dose‐adjusted prescriptions for bicalutamide, abiraterone, enzalutamide, apalutamide, darolutamide, and other vintages (estramustine phosphate, flutamide) was 276, 14.2, 18.1, 2.19, 0.34, and 20.3 million, respectively. The usage of ARSI increased significantly from 6.1% in 2016 to 16% in 2021. The medical costs for prostate cancer‐specific agents increased significantly (1.8‐fold) from 2016 to 2021. Despite the limited usage of ARSIs, a majority of the medical costs had been spent on ARSIs. Medical costs associated with ARSIs increased significantly from 59% to 89% (*p* < 0.001).

**Conclusion:**

ARSI usage and medical costs associated with prostate cancer increased significantly from 2016 to 2021. Despite the limited use of ARSIs, a considerable proportion of the medical costs for prostate cancer‐specific agents had been spent on ARSIs.

## Graphical abstract

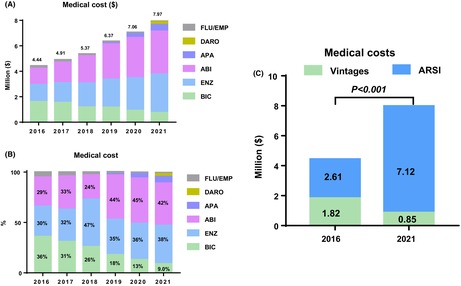

## 1 INTRODUCTION

Prostate cancer (PC) remains the most common malignant disease in men worldwide.^1^, ^2^ However, the introduction of novel antiandrogen receptor signal inhibitors (ARSIs) has significantly changed the treatment strategy for metastatic castration‐sensitive prostate cancer (mCSPC) and castration‐resistant prostate cancer (CRPC).^3^ Indeed, studies have shown that abiraterone acetate (ABI), enzalutamide (ENZ), apalutamide (APA), and darolutamide (DARO) significantly improved oncological outcomes in patients with CRPC.^4^, ^5^, ^6^, ^7^ Furthermore, upfront intensification therapies for mCSPC consisting of androgen deprivation therapy plus ARSIs have been developed through pivotal clinical trials^8^, ^9^, ^10^, ^11^ and real‐world practice.^3^, ^12^, ^13^, ^14^ This dramatic paradigm shift has promoted a significant change in the standard treatment of mCSPC and CRPC. However, the tradeoff for the efficacy of ARSIs has been the increase in the cost of medical care associated with use of expensive agents.^15^, ^16^, ^17^ The use of ARSIs as upfront intensification therapies prolongs OS but further increases the duration of agent use and long‐term medical costs. Given the promising clinical benefits of ARSI upfront intensification therapies, characterizing real‐world treatment trends and medical costs in affected patients is warranted. However, no report has yet examined the economic burden placed on overall healthcare system. We retrospectively evaluated trends in ARSI use and medical costs in patients with prostate cancer using The National Database of Health Insurance Claims and Specific Health Checkups of Japan (NDB) Open Data, which covers almost all clinical practice (>95%) throughout Japan.^18^

## 2 METHODS

Given that the current study analyzed publicly available data, no ethics approval was requested. The NDB Open Data office requested that published data be submitted to them.

To identify the patients who were treated with vintage antiandrogen therapy (bicalutamide [BIC], flutamide [FLU], estramustine phosphate [EMP]) or ARSI (ABI, ENZ, APA, and DARO), we analyzed data available in the NDB Open Data from 2016 to 2021, which had been published by the Ministry of Health, Labor and Welfare. Given that the NDB Open Data only provides information on the number of prescribed tablets, the number of people covered remained not known. Therefore, we calculated the number of person‐days (person × prescription days) based on a standard dosage. Standard doses were set as follows: ABI (250 mg, 4 tablets), ENZ (40 mg, 4 tablets), APA (60 mg, 4 tablets), DARO (300 mg, 4 tablets), EMP (156.7 mg, 4 capsules), FLU (125 mg, 3 tablets), ENZ (80 mg, 2 tablets), BIC (80 mg, 1 tablet).

The primary objective was to determine the annual trends in ARSI usage and medical costs. The secondary objective was to determine the trends of prescriptions according to age group. The exchange rate was calculated at 140 Japanese yen to the dollar.

In Japan, the use of ARSIs (ABI or ENZ) for CRPC treatment has been covered by the national insurance system since 2014. Upfront ABI for LATITUDE high‐risk disease and upfront ENZ/APA for all‐risk groups had been approved in February 2018 and May 2020, respectively. The use of darolutamide for nonmetastatic CRPC was approved in Japan in January 2020. The choice of therapeutic agent is determined by the attending physician. Patients who developed metastatic castration‐resistant PC were treated with sequential therapy, including ARSI or taxane‐based chemotherapy.

Statistical analyses were performed using GraphPad Prism 7.00 (GraphPad Software, San Diego, CA, United States) and BellCurve for Excel (Social Survey Research Information Co., Ltd., Tokyo, Japan). Categorical variables were compared using the Fisher exact test. *p* < 0.05 indicated statistical significance.

## 3 RESULTS

Between 2016 and 2021, the NDB Open Data included a total of 459,610 million tablets/capsules prescribed. Among these, standard dose‐adjusted prescriptions for BIC (275.9 million), FLU and EMP (20.25 million), and ARSI (8.70 million) were selected (Table 1).

**TABLE 1:** Number of prescriptions and medical costs between 2016 and 2021.

Prescriptions (million tablets/capsules)	2016	2017	2018	2019	2020	2021
BIC	45.5	45.5	44.8	46.4	46.7	47.0
FLU/EMP	3.95	3.76	3.46	3.26	3.08	2.75
ENZ	1.99	2.31	2.83	3.34	3.82	4.5
ABI	1.2	1.54	2.03	2.63	2.94	3.13
APA	‐	‐	‐	0.12	0.46	1.61
DARO	‐	‐	‐	‐	‐	0.34
Medical costs (million $)	2016	2017	2018	2019	2020	2021
BIC	1.61	1.54	1.19	1.17	0.92	0.75
FLU/EMP	3.95	3.76	3.46	3.26	3.08	2.75
ENZ	1.34	1.55	1.88	2.2	2.56	3.02
ABI	1.27	1.62	2.14	2.77	3.16	3.37
APA	‐	‐	‐	0.08	0.3	0.52
DARO	‐	‐	‐	‐	‐	0.22

Abbreviations: ABI, abiraterone; APA, apalutamide; BIC, bicalutamide; DARO, darolutamide; EMP, estramustine phosphate; ENZ: enzalutamide.

**FIGURE 1:**
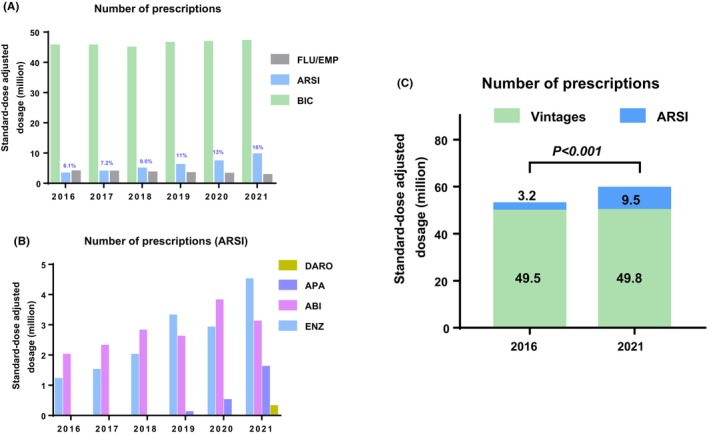
Trends in prescriptions. (A) Number of prescriptions for bicalutamide (BIC), flutamide (FLU), and estramustine phosphate (EMP) and novel antiandrogen receptor signal inhibitors (ARSI) between 2016 and 2021. B: Number of prescriptions for ARSIs (abiraterone [ABI], enzalutamide [ENZ], apalutamide [APA], darolutamide [DARO]). C: Comparison of the number of prescriptions between 2016 and 2021.

Trends in the prescription rate of ARSIs increased from 6.1% in 2016 to 16.0% in 2021 (2.6‐fold), whereas trends in the prescription rate of vintages (BIC/FLU/EMP) remained stable (Figure 1A). Trends in ARSI prescription are shifting from ABI‐dominated to APA/ENZ (Figure 1B). ARSI use was significant greater in 2021 than in 2016 (Figure 1C).

**FIGURE 2:**
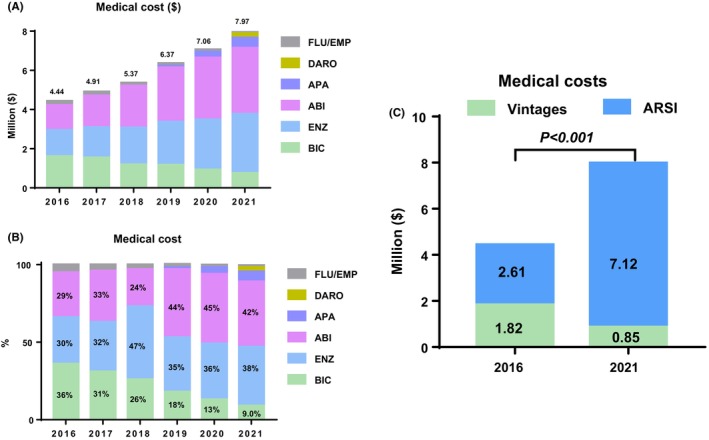
Trends in medical costs. (A): Trends in medical costs associated with prostate cancer‐specific agents between 2016 and 2021. The exchange rate was calculated at 140 Japanese yen to the dollar. (B) Trends in the proportion of medical cost between 2016 and 2021. (C) Comparison of medical costs between 2016 and 2021. BIC, FLU, and EMP were classified as vintage therapy.

Medical costs for prostate cancer agents have been increasing every year, from 4.44 million $ in 2016 to 7.97 million $ in 2021 (1.8‐fold) (Figure 2A). The proportion of ARSI cost increased from 59% in 2016 to 89.8% in 2021 (Figure 2B). Medical costs associated with ARSIs were significantly greater in 2021 than in 2016 (Figure 2C).

**FIGURE 3:**
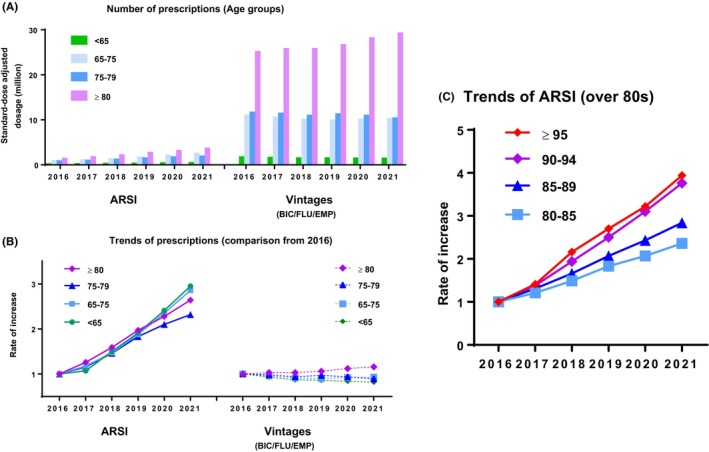
Trends in prescriptions according to age group. (A) The number of ARSI (abiraterone [ABI], enzalutamide [ENZ], apalutamide [APA], darolutamide [DARO]) and vintage (bicalutamide [BIC], flutamide [FLU], and estramustine phosphate [EMP]) prescriptions according to age group. (B) Comparison of prescriptions based on 2016 data. (C) Comparison of ARSI prescriptions in patients over 80 based on 2016 data.

Trends in prescriptions according to age group are summarized in Figure 3A. Accordingly, our findings showed an increase in prescription of ARSI but not vintages across all ages while (Figure 3B). ARSI prescriptions was increased in the group of patients over 80 years of age. Our results showed that older patients had higher rates of increase (Figure 3C). ARSI use in 2021 was 2.36‐, 2.84‐, 3.76‐, and 3.94‐folds higher that than in 2016 for patients aged 80–85, 85–90, 90–94, and ≥95 years, respectively.

## 4 DISCUSSION

The current study has been the first to the analyze trends in prostate cancer‐specific agent use and costs using real‐world big data covering most of the clinical practices throughout Japan. Although ARSIs have been found to improve prognosis, the associated medical costs can be considered a disadvantage. Considering this balance, this study provides important foundational findings.

First, at a usage rate of 6.1% (in 2016), ARSIs accounted for more than half of the prostate cancer medication costs. As upfront treatment became more widespread, ARSI usage increased, reaching 89% of the prostate cancer medication costs in 2021 despite having a usage rate of only 16.0%. Conversely, a decrease in the medical cost had been observed with vintage agents (e.g., bicalutamide) following the introduction of generic agents. The introduction of generic ARSI in Japan is also awaited.

Second, ARSI use increased among elderly patients. Notably, our findings showed that older patients, even those in ≥90 years old, exhibited an increase in usage rates. Although distinguishing between mCSPC and CRPC is not possible using information obtained from the NDB Open Data, this may be an increased opportunity to use ARSIs as upfront intensive therapies. A prior retrospective study reported that ARSIs were effective for mCSPC in patients over 75 years of age^19^; however, the efficacy for very elderly patients over 80 years of age has been unknown. A large Swedish cohort found overall survival benefits from upfront intensive therapy in those over 80 years of age, although the overall population did show an improvement in overall survival.^20^ This indicates that the benefits of upfront intensification therapy are limited in very elderly patients with a short life expectancy. Thus, the efficacy of upfront ARSI for patients older than 80 years remains questionable. Further studies would be needed to determine the appropriate cutoff age that balances the risks and benefits.

It is clear that the cost of ARSI is a problem, especially in less developed countries. A survey from 16 specialists on clinical practice patterns from 7 Southeast Asia countries for advanced PC care showed that the cost is one of the important factors influencing therapeutic decisions.^21^ Of those, 94% of panelists answered that androgen deprivation therapy is the primary systemic treatment for advanced PC. All panelists supported the use of generic versions of approved therapies. Cost‐effectiveness of ARSI is also a major issue in India.^22^ Furthermore, it is the problem in developed countries without adequate public insurance. A survey of search popularity in the United States using Google Trends™ also reported that bicalutamide was more frequently searched than ARSI among lower household income and those without medical insurance.^23^ We have to wait until generic agents become available. The launch of generic medications may shed some light on this issue.

Some limitations of the current study include the lack of individual data, the number of accurate prescriptions, and the treatment status (mCSPC or CRPC). First, it is difficult to obtain the amount of mCSPC and CRPC used separately from the NDB. Furthermore, modifying the daily dose may results in inaccurate person‐day values. Second, it is not possible to make comparisons with other countries/regions because we only analyzed data from within Japan. The difficulty with this issue is that the rules are different in each country and region. For example, only one 2‐year ARSI per lifetime is allowed in Taiwan. It means that only one agent can be used through mCSPC/mCRPC treatment. Differences in health care systems from country to country makes difficult simple comparisons. Third, we were unable to compare urban and rural areas in our study because this database does not include regional information within Japan. There may be a difference in the usage rate of ARSI between urban and rural areas. Data from our multicenter study indicate that there is a significant difference in the frequency of ARSI usage depending on whether the patient was treated at an academic center (86%) or a community hospital (35%). Future research will be needed to compare urban and rural areas across Japan. Fourth, it is necessary to consider the balance between OS profits and costs in the future. Fifth, we could not address quality‐adjusted life years and incremental cost‐effectiveness ratio due to the lack of information. However, a major strength of our study was that we were able to clarify the current trends in ARSI use and medical costs via big data covering more than 95% of clinical practices throughout Japan. Our next study needs to address those issues.

## 5 CONCLUSIONS

ARSI usage and medical costs for prostate cancer increased significantly from 2016 to 2021. Despite the limited use of ARSIs, the majority of medical costs for prostate cancer‐specific agents were spent on ARSIs.

## AUTHOR CONTRIBUTIONS

**Hikari Miura:** Data curation (equal); writing – original draft (equal); writing – review and editing (equal). **Hayato Yamamoto:** Data curation (equal). **Yoshiharu Okuyama:** Data curation (equal). **Noritaka Ishi:** Data curation (equal). **Ryuma Tanaka:** Data curation (equal). **Takuya Oishi:** Data curation (equal). **Fumiya Yoneyama:** Data curation (equal). **Tomoko Hamaya:** Data curation (equal). **Kyo Togashi:** Data curation (equal). **Naoki Fujita:** Data curation (equal); investigation (equal); resources (equal); writing – review and editing (equal). **Teppei Okamoto:** Data curation (equal); writing – review and editing (equal). **Chikara Ohyama:** Conceptualization (equal); funding acquisition (equal); resources (equal); supervision (equal). **Shingo Hatakeyama:** Conceptualization (equal); data curation (equal); formal analysis (equal); funding acquisition (equal); investigation (equal); methodology (equal); project administration (equal); resources (equal); software (equal); writing – original draft (equal); writing – review and editing (equal).

## FUNDING INFORMATION

The authors have no funding to declare.

## CONFLICT OF INTEREST STATEMENT

The authors declares no conflicts of interest.

## ETHICS STATEMENT

No ethical issues with publicly available open data.

## Data Availability

The data that support the findings of this study are available from the corresponding author upon reasonable request.
